# Relation of amino acid composition, hydrophobicity, and molecular weight with antidiabetic, antihypertensive, and antioxidant properties of mixtures of corn gluten and soy protein hydrolysates

**DOI:** 10.1002/fsn3.3160

**Published:** 2022-12-05

**Authors:** Homaira Mirzaee, Hassan Ahmadi Gavlighi, Mehdi Nikoo, Chibuike C. Udenigwe, Faramarz Khodaiyan

**Affiliations:** ^1^ Department of Food Science and Technology, Faculty of Agriculture Tarbiat Modares University Tehran Iran; ^2^ Institute for Natural Products and Medicinal Plants Tarbiat Modares University Tehran Iran; ^3^ Department of Pathobiology and Quality Control, Artemia and Aquaculture Research Institute Urmia University Urmia Iran; ^4^ School of Nutrition Sciences University of Ottawa Ottawa Ontario Canada; ^5^ Bioprocessing and Biodetection Laboratory, Department of Food Science and Engineering University of Tehran Karaj Iran

**Keywords:** bioactivities, corn protein, functional properties, hydrolysates, soy protein

## Abstract

New mixed Alcalase‐hydrolysates were developed using corn gluten meal (CP) and soy protein (SP) hydrolysates, namely CPH, SPH, SPH30:CPH70, SPH70:CPH30, and SPH50:CPH50. Amino acid profile, surface hydrophobicity (*H*_0_), molecular weight (MW) distribution, antioxidant activity, angiotensin‐converting enzyme (ACE), α‐amylase, and α‐glucosidase inhibitory activities, and functional characteristics of hydrolysates were determined. Hydrolysis changed the amount of hydrophilic and hydrophobic amino acid composition and significantly increased the *H*_0_ values of hydrolysates, especially for CPH. The DPPH radical scavenging activity (RSA) was higher for CPH, SPH30:CPH70, and SPH50:CPH50 than SPH and SPH70:CPH30. Moreover, SPH, SPH70:CPH30, and SPH50:CPH50 showed lower MW than CPH, and this correlated with the higher hydrophilicity, and ABTS and hydroxyl RSA values obtained for SPH and the mixed hydrolysates with predominantly SPH. SPH70:CPH30 exhibited higher ACE, α‐glucosidase, and α‐amylase inhibitory activities among all samples due to its specific peptides with high capacity to interact with amino acid residues located at the enzyme active site and also low binding energy. At 15% degree of hydrolysis, both SPH and CPH showed enhanced solubility at pH 4.0, 7.0 and 9.0, emulsifying activity, and foaming capacity. Taken together, SPH70:CPH30 displayed strong antioxidant, antihypertensive, and antidiabetic attributes, emulsifying activity and stability indexes, and foaming capacity and foaming stability, making it a promising multifunctional ingredient for the development of functional food products.

## Graphical abstract

In this study, new formulation of mixed Alcalase‐hydrolysates was developed using corn gluten meal (CP) and soy protein (SP) hydrolysates, namely CPH, SPH, SPH30:CPH70, SPH70:CPH30, and SPH50:CPH50. Amino acid profile, surface hydrophobicity (H0), molecular weight (MW) distribution, antioxidant activity, angiotensin‐converting enzyme (ACE) and antidiabetic activities, and functional characteristics of hydrolysates were determined. For CPH, protein or peptide bands with MW of 30–40, 50–55, 70–80, and 150–170 kDa were observed. For SPH, most of the bands at 70–200 kDa assigned to SP disappeared, and new bands were formed at 10–15, 15–20, 30, 40–50, and 50–60 kDa. SPH70:CPH30 revealed higher ACE, a‐glucosidase, and a‐amylase inhibitory activities among all samples. At 15% degree of hydrolysis, both SPH and CPH showed enhanced solubility at pH 3.0, 7.0, and 9.0, emulsifying activity, and foaming capacity. Taken together, SPH70:CPH30 with high antioxidant, antihypertensive, and antidiabetic attributes. These results have important implications for the designing of mixed protein hydrolysate as by‐product used for the development of novel antidiabetic, antihypertensive, and antioxidant additives for food industrial application.

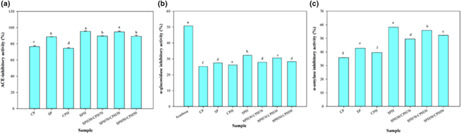

## 1 INTRODUCTION

Plant proteins have been widely considered as sustainable ingredients for the development of bioactive peptides and hydrolysates with antidiabetic, antihypertensive, and antioxidant activities (Das et al., 2022; Jin, Liu, et al., 2016). Enzymatic hydrolysis is a mild process that does not damage the amino acid composition compared with acidic hydrolysis, which could break down amino acids to toxic substances, such as 3‐chloropropane‐1,2‐diol with carcinogenic effects (Adler‐Nissen, 1979, 1984; Lee & Khor, 2015; Nikoo et al., 2022; Wong et al., 2020). Thus, enzymatic hydrolysis is widely used in food industry as a controllable process for limited hydrolysis of proteins (Rezvankhah et al., 2021a, 2021b; Rezvankhah, Yarmand, et al., 2022).

The activity of α‐glucosidase and α‐amylase is associated with diabetes mellitus (Chandrasekaran & Gonzalez de Mejia, 2022; de Matos et al., 2022; Jiang et al., 2018; Qiao et al., 2020). These enzymes, secreted by the pancreas, break down dietary disaccharides and polysaccharides (Fadimu, Gill, et al., 2022; Karimi et al., 2020). The resulting glucose is absorbed at a higher rate into the blood, resulting in increased blood glucose level (Chandrasekaran & Gonzalez de Mejia, 2022; Tacias‐Pascacio et al., 2020). Moreover, hypertension is associated with angiotensin I‐converting enzyme (ACE), an enzyme of the renin‐angiotensin system pathway and important target of antihypertensive agents (Gharibzahedi & Smith, 2021; Guo et al., 2020; Ozón et al., 2022; Wang et al., 2019). Plant protein hydrolysates have shown strong inhibitory activities against α‐glucosidase, α‐amylase, and ACE toward the prevention and management of diabetes and hypertension (Karimi et al., 2020, 2021; Liu et al., 2020; Qiao et al., 2020). These inhibitory activities have been attributed to the presence of specific peptides, predominantly those composed of highly hydrophobic amino acids, released by commercial proteases (Das et al., 2022). The interaction between these peptides and amino acid residues at the active site of enzymes leads to inhibition of enzymatic activity (Quintero‐Soto et al., 2021). Strong ACE‐inhibitory activity has been reported for lentil protein hydrolysates obtained from sequential hydrolysis with Alcalase and Flavourzyme (Rezvankhah et al., 2021a, 2021b). In addition, cross‐linked lentil protein hydrolysates showed improved ACE‐inhibitory activity (Rezvankhah, Emam‐Djomeh, et al., 2022; Rezvankhah, Yarmand, et al., 2022).

Corn gluten meal (CGM) is a major by‐product of the corn wet milling process (Liu et al., 2020). It contains 62%–71% protein with zein as the prominent protein fraction, accounting for 68% of the total protein, and glutelin as the residual part (~28% of zein weight) (Hu et al., 2022; Ren et al., 2018). Zein limits the application of CGM in various foods due to its poor water solubility (Yang et al., 2007). Furthermore, CGM contains several hydrophobic amino acid residues, which are buried inside the protein structure (Shen et al., 2020). Corn gluten meal is deficient in lysine and tryptophan, limiting its use in human nutrition (Zhu et al., 2019). Some studies have reported that enzymatic modification of CGM improved its solubility and bioavailability (Jiang et al., 2020; Jin, Liu, et al., 2016; Jin, Ma, et al., 2016; Liu et al., 2015). Corn protein hydrolysates (CPH) consist of small peptides with different molecular weight (MW) profiles, including di‐ and tripeptides, which can be effectively absorbed into blood circulation (Jin, Liu, et al., 2016; Jin, Ma, et al., 2016). Antioxidant and ACE‐inhibitory activities of CPH have been reported (Li et al., 2019; Ren et al., 2018; Wang et al., 2020; Yang et al., 2007). When subjected to gastrointestinal digestion, CPH showed 12.9% increased antioxidant activity while retaining 77.5% of peptides compared with the undigested hydrolysates (Ren et al., 2018). Moreover, Yang et al. (2007) reported that peptide Ala‐Tyr in Alcalase‐hydrolyzed CGM at 50 mg/kg body weight decreased the systolic blood pressure of rats by 9.5 mmHg at 2 h after oral administration. Furthermore, corn germ protein hydrolysates (CGPH) and associated peptidic fraction (F1) with MW <2 kDa showed higher radical scavenging and α‐glucosidase inhibitory activities than F2 fraction with MW of 2–10 kDa (Karimi et al., 2020). These inhibitory activities can be attributed to the different amino acid sequences, which determine the interactions with the active site residues of the enzyme (Quintero‐Soto et al., 2021).

Soy proteins (SPs) are one of the most utilized plant proteins in foods due to their nutritional quality, availability, and affordability compared with other sources of plant proteins (Xu et al., 2021). Native SPs are composed of a mixture of globulins and albumins (Tian et al., 2020). Ninety percent of the proteins are storage proteins with a globular structure consisting mainly of 7S (β‐conglycinin) and 11S (glycinin) globulins (Chen et al., 2011a, 2011b). Soy proteins have been considered for their emulsifying activity and gelling potential compared with other plant proteins (Bessada et al., 2019). However, native SP has compact globular structures, leading to low molecular flexibility, relatively low emulsifying properties, and antioxidant activities compared with its modified states (Zhang et al., 2021). Moreover, dietary SPs have shown antidiabetic activity in humans, indicating potential involvement of α‐amylase and α‐glucosidase inhibition (Das et al., 2022; Wang et al., 2019; Zhang et al., 2021; Zhao et al., 2021). Enzymatic hydrolysis of SP has been shown to increase solubility, antioxidant and α‐glucosidase inhibitory, and antihypertensive activities of SPH (Jiang et al., 2018; Tian et al., 2020; Wang et al., 2019). α‐Glucosidase inhibitory activity of SPH was reported to be higher than that of flaxseed, rapeseed, sunflower, and sesame protein hydrolysates (Han et al., 2021). Furthermore, novel peptides IY, YVVF, LVF, WMY, LVLL, and FF were identified from ACE‐inhibiting Alcalase‐derived SPH (Xu et al., 2021). The high hydrophobicity scores of the peptides might be the main contributor to the activity of SPH. The C‐terminal hydrophobic residues showed important interactions that may have contributed to ACE inhibition (Xu et al., 2021).

A combination of plant protein hydrolysates can compensate for the deficiency of individual hydrolysates (Akharume et al., 2021). For instance, CGM has a low amount of lysine but is rich in methionine and cysteine and hydrophobic amino acids (Hu et al., 2022). Conversely, SP has a high content of lysine but limited sulfur‐containing and hydrophobic amino acids (Tian et al., 2020). The combination of hydrolysates not only improves the deficiencies and balances the amino acid composition but also can augment biological (i.e., antioxidant, antidiabetic, and antihypertensive activities), and functional properties such as emulsifying activity. Since CGM and CPH demonstrate weaker ACE inhibitory activity than SP and SPH, it is postulated that combinations of SPH and CPH can produce improved hypertension property (Xu et al., 2021).

Hence, the objective of this study was to investigate the influence of combinations of CGM and SP hydrolysates on the bioactivities, including in vitro antidiabetic, antihypertensive and antioxidant activities, and functional properties. There is a dearth of studies on the combination of complementary bioactive hydrolysates; thus, the mixed protein hydrolysates can be explored as novel plant‐based ingredients with augmented antioxidant, antihypertensive, antidiabetic, and techno‐functional properties.

## 2 MATERIALS AND METHODS

### 2.1 Materials

Soy protein isolate (SPI, ~90% protein) and CGM (~62% protein) were supplied by Shansong Industrial Chinese Co. Ltd. and a grain processing refinery, Golshahd Co. Ltd., respectively. Alcalase 2.4 L from *Bacillus licheniformis*, with the activity of 2.4 Anson Units (AU)/g, and a density of 1.18 g/ml was purchased from Novozymes. 2,2 Diphenyl‐1‐picrylhydrazyl (DPPH), 2,2′‐azino‐bis (3‐ethylbenzthiazoline‐6‐sulphonic acid) diammonium salt (ABTS), 4‐nitrophenyl *α*‐d‐glucopyranoside (PNPG), porcine pancreatic α‐amylase, rat intestinal α‐glucosidase, ACE (5 UN), hippuryl‐his‐leu (HHL), and ammonium salt of 1‐anilino‐8‐naphtalene‐sulphonic acid (ANS) were purchased from Sigma‐Aldrich. Soluble starch ACS reagent was purchased from Merck.

### 2.2 Enzymatic hydrolysis of proteins

Protein solutions of SPI (~90% protein) and CGM (~62% protein) were prepared at 5% w/w. Soy protein isolate solution was heated at 90°C for 15 min to unfold the protein while the CGM solution was heated at 100°C for 30 min due to its low water solubility, thus the intense thermal treatment for denaturation (Tian et al., 2020; Yang et al., 2007). Enzymatic hydrolysis using Alcalase was conducted at temperature 60°C, pH 8.0, and enzyme/substrate ratio of 2.5% w/w. This condition was optimized in preliminary studies. Hydrolysis time was considered based on DH reaching 15%; thus, 90 and 210 min were obtained for hydrolysis of SPI and CGM, respectively. Corn gluten meal have zein and glutelin as the main proteins (heat resistant) and thus required longer hydrolysis time to reach DH of 15% (Yang et al., 2007). After the reaction, CPH and SPH solutions were heated at 95°C for 10 min to terminate enzymatic activity. Then, the protein/peptide solutions were centrifuged at 15,000 *g* for 10 min and the obtained supernatants (rich in soluble peptides) were collected and adjusted to pH 7.0 using 1 M HCl, and spray‐dried (DORSA tech) through a drying air of 180°C (the exhausting temperature of 75–80°C) and an air flow of 0.3–0.4 MPa (Akbarbaglu et al., 2019; Rezvankhah, Emam‐Djomeh, et al., 2022; Rezvankhah, Yarmand, et al., 2022; Sarabandi et al., 2019). The powdered hydrolysates were stored at −18°C until the next experiments.

### 2.3 Determination of the degree of hydrolysis (DH)

Degree of hydrolysis was determined using the pH‐stat protocol reported by Adler‐Nissen (1986) and calculated using the equation:

(1) $$\mathrm{DH}\;\left(\%\right)=B\times N_{b}\times \frac{1}{\alpha }\times \frac{1}{M_{p}}\times \frac{1}{h_{\mathrm{tot}}}$$

where *B* is the volume (ml) of NaOH needed to maintain the pH constant; *N*_b_ is the normality of the consumed base; *M*_p_ is the mass of protein in CP and SP; *h*_tot_ is the total number of peptide bonds in the protein substrates (considered 9.2 for CP and 7.75 for SP) (Adler‐Nissen, 1979, 1986; Jin et al., 2015; Tian et al., 2020; Xu et al., 2021); and *α* is the amount of α‐NH_2_ released during the proteolysis reaction.

### 2.4 Preparation of mixture hydrolysates

CPH and SPH were mixed homogeneously using a mixer (Moulinex, LM238125) at three proportions, including 30:70, 50:50, and 70:30 (% w/w), which were referred to as SPH30:CPH70, SPH70:CPH30, and SPH50:CPH50, respectively. Unhydrolyzed proteins (CP and SP) were used as control in all analyses.

### 2.5 Amino acid composition

The amino acid profiles were determined using reversed‐phase high‐performance liquid chromatography (RP‐HPLC, Agilent 1100 HPLC; Agilent Ltd.), as described by Liu et al. (2012). First, the samples were hydrolyzed in the glass tubes using 6 M HCl at 120°C for 12 h. Thereafter, the digests were filtered through 0.22 μm pore size filter. The separation was performed using a Zorbax analytical column (C18, 4 × 250 mm, 5 μm particle size; Agilent) at the temperature of 40°C with a UV detector spectra monitored at 338 nm. The elution of column with the flow rate of 1 ml/min was conducted with mobile phases comprising 7.40 mmol/L of sodium acetate/triethylamine/tetrahydrofuran (400:0.10:2, v/v/v), set at pH 7.1 using acetic acid and 7.40 mmol/L of sodium acetate/methanol/acetonitrile (1.5:2.5:2.5, v/v/v), set at pH 7.1. A standard solution comprising of 17 amino acids was used as an external standard.

### 2.6 Surface hydrophobicity ( *H*_0_ )

The protein and hydrolysate samples were investigated for the *H*_0_ using 1‐anilinonaphthalene‐8‐sulfonic (ANS) according to the method of He et al. (2021). The samples were diluted to 0.01–0.02 mg/ml in 10 mM phosphate buffer (pH 7). The fluorescence intensity was measured at a wavelength of 390 nm (excitation) and 470 nm (emission) using a fluorescence spectrophotometer (LS‐55, Perkin Elmer). The slope of the plot of fluorescence vs. concentration of samples was expressed as the surface hydrophobicity (*H*_0_).

### 2.7 SDS‐polyacrylamide gel electrophoresis (SDS‐PAGE)

SDS‐polyacrylamide gel electrophoresis was used to estimate the MW profile of CP, SP, and their hydrolysates following the method of Rezvankhah et al. (2021b). Briefly, a sample solution (5 mg/ml) of proteins and respective hydrolysates was mixed with an equal amount of Laemmli sample buffer (960 μl of 66 mM Tris–HCl, pH 6.9, 27.3% w/v glycerol, 2.2% SDS, 0.01% bromophenol blue). Then, the prepared samples were combined with 2‐mercaptoethanol and heated for denaturation at 95°C for 5 min before the electrophoresis. The concentration of 12% Mini‐Protean™ precast gels (Bio‐Rad) was used to run the electrophoresis. Thereafter, 10 μl of cooled samples was loaded on the gels, and then subjected to a constant voltage of 150 V. Additionally, a marker with MW standards (Bio‐Rad Broad Range Marker) was run alongside the samples. When the process finished, the gels were stained with 0.1% Coomassie Brilliant Blue R‐250 in a mixture of 10% acetic acid and 40% methanol for 2 h. The protein/peptide bands were visualized by discoloring the gels using a mixture of 40% methanol and 10% acetic acid solutions.

### 2.8 MW distribution

Gel permeation chromatography (GPC) was applied to determine MW distribution of the CP, SP, and hydrolysate samples following the method of Rezvankhah et al. (2021b). The Waters Breeze HPLC system (Waters Corporation) equipped with a Waters UV detector and Superdex Peptide HR column (30 cm × 10 mm and 13–15 μm particle size) was used to evaluate the protein/peptide sizes. The analytes were dissolved in ultrapure water and stirred for 30 min at 25°C. The solution was centrifuged at 12,000 *g* for 10 min, and the supernatant was filtered through a 0.22 μm membrane filter. The injection volume of sample was 50 μl, and the column was eluted with 20 mM phosphate buffer containing 0.15 M NaCl (pH 7) at a flow rate of 0.5 ml/min. The spectra were monitored at a wavelength of 210 nm. Standard compounds of known MW including reduced glutathione (300 Da), glutathione disulfide (600 Da), cyanocobalamin (1355 Da), aprotinin (6500 Da), and cytochrome C (12,500 Da) were used to prepare a standard curve, which was used to determine the MW.

### 2.9 Antioxidant activity

#### 2.9.1 DPPH radical scavenging activity

Antioxidant activity was determined according to the method of Zheng et al. (2015). 2,2 Diphenyl‐1‐picrylhydrazyl solution at 0.2 mM in 95% ethanol and the protein/peptide solution at 7 mg/ml were prepared. Then, 2 ml of DPPH solution was combined with 2 ml of samples and stored for 30 min in the dark. The absorbance of the mixtures was read at 517 nm using a spectrophotometer. To compare the antioxidant activity of the analytes, ascorbic acid (0.01 mg/ml) was used as a positive control. The RSA% was calculated using the equation:

(2) $$\mathrm{RSA}\;\left(\%\right)=\frac{A_{C}-A_{S}}{A_{C}-A_{B}}\times 100$$

where the absorbance values were for control (*A*_C_), sample (*A*_S_), and blank (*A*_B_).

#### 2.9.2 ABTS radical cation scavenging activity

The ABTS^·+^ scavenging activity of hydrolysates was evaluated according to the protocol reported by Amini Sarteshnizi et al. (2021). The ABTS solution (940 μl) was combined with 60 μl of samples (7 mg/ml) and vigorously shaken, and then incubated at 25°C for 10 min in the dark. The absorbance was read at 734 nm using a spectrophotometer, and ascorbic acid (0.01 mg/ml) was used as a positive control.

#### 2.9.3 Hydroxyl radical scavenging activity (RSA)

The hydroxyl RSA was evaluated using the protocol reported by Zheng et al. (2015). Two milliliter of samples (7 mg/ml), 2 ml of FeSO_4_ (6 mM), and 2 ml of H_2_O_2_ (6 mM) were thoroughly mixed and kept for 10 min at room temperature. Then, 2 ml of salicylic acid (6 mM) was added and incubated for 30 min. The absorbance was then read at 510 nm (*A*_S_). Distilled water was used as the blank (instead of salicylic acid solution) and the control (instead of sample solution). Ascorbic acid (0.01 mg/ml) was used as a positive control. The RSA was calculated using equation 2.

### 2.10 ACE inhibition assay

The potential antihypertensive activity of CP, SP, and hydrolysates was assessed by the in vitro inhibition of angiotensin I‐converting enzyme (ACE) based on the method of Boye et al. (2010). ACE inhibition (%) was computed by the equation:

(3) $$\text{Inhibitory activity}\;\left(\%\right)=\frac{A_{C}-A_{S}}{A_{C}-A_{B}}\times 100$$

where the absorbance values were for control (*A*_C_), sample (*A*_S_), and blank (*A*_B_). Also, the IC_50_ value, the concentration of sample that inhibited 50% of ACE activity, was determined using sample concentrations of 0.1–2 mg/ml.

### 2.11 Determination of in vitro antidiabetic properties

#### 2.11.1 α‐Glucosidase inhibition assay

The inhibitory activity of CP, SP, and their hydrolysates against rat intestinal α‐glucosidase was assessed following the method of Karimi et al. (2020). Briefly, the enzyme was extracted from acetone powder from rat intestine, and the obtained solution was diluted to 90 mU/ml. Then, 150 μl of different concentrations of the samples (10–500 μg/ml) was combined with 250 μl of α‐glucosidase and incubated at 37°C for 10 min. To carry on the reaction, 100 μl of PNPG solution (5 mM) was added and the mixture was incubated at 37°C for 30 min while scanning the absorbance at 405 nm every 2 min. Instead of analyte solution, phosphate buffer was utilized as a control. To compare the inhibitory activity, acarbose (0.5 mg/ml), a synthetic antidiabetic compound, was used as a positive control. The enzyme inhibition was calculated using the equation:

(4) $$\text{Inhibtion of}\;\alpha -\text{glucosidase}\;\left(\%\right)=\frac{A_{C}-A_{S}}{A_{C}}\times 100$$

where the absorbance values were for control (*A*_C_) and sample (*A*_S_). Sample concentrations of 10–500 μg/ml were used to determine the IC_50_ values.

#### 2.11.2 α‐Amylase inhibition assay

The α‐amylase inhibitory activity of CP, SP, and hydrolysates was determined using the method reported by Rahimi et al. (2022). Briefly, 100 μl of different concentrations of the samples (10–500 μg/ml) was combined with 120 μl of α‐amylase solution (0.6 U/ml) and incubated at 37°C for 5 min. Then, 120 μl of 0.5% (w/v) starch solution was added. The enzyme activity was terminated by heating the reaction mixture at 100°C for 10 min followed by cooling to ambient temperature. The undigested starch was separated by centrifugation at 15,000 *g* for 2 min. Then, 20 μl of the supernatant was mixed with 1 ml of PAHBAH and the solution was heated to 70°C for 10 min. The sample solutions were cooled, and absorbance values were read at 410 nm. The inhibitory activity was determined using the following equation:

(5) $$\text{Inhibition of}\;\alpha -\text{amylase}\;\left(\%\right)=\left(1-\frac{\left(A_{S}-A_{B}\right)}{A_{C}}\right)\times 100$$

where the absorbance values were for sample (*A*_S_), blank (*A*_B_, phosphate buffer, enzyme, sample), and control (*A*_C_, starch, buffer, enzyme). Furthermore, IC_50_ values were determined as previously described. Acarbose, at its IC_50_ value (0.125 mg/ml), was used as a positive control.

### 2.12 Determination of functional properties

#### 2.12.1 Solubility

The solubility of CP, SP, and hydrolysates was assessed by the method of Fathollahy et al. (2021) with slight modifications. Briefly, 10 mg/ml of samples at three pH values (4.0, 7.0, and 9.0) was centrifuged at 8000 *g* for 20 min. The supernatants were taken for protein determination based on the Bradford protocol (Bradford, 1976). To prepare the standard curve and calculate the protein content, bovine serum albumin was used as a reference protein. The solubility was determined by the following equation:

(6) $$\text{Solubility}\;\left(\%\right)=\frac{\text{Protein content in the supernatant}\;}{\text{Total protein content in the sample}}\times 100$$

#### 2.12.2 Emulsifying properties

Two emulsifying properties including emulsifying activity index (EAI, m^2^/g) and emulsifying stability index (ESI, min) were assessed by the method of Rezvankhah et al. (2021b). Samples (10 mg/ml) were prepared and combined with 1 ml of sunflower oil and homogenized at 19,000 rpm for 1 min using a laboratory‐scale homogenizer (IKA, T25). The emulsion (100 μl) was taken from the container bottom immediately after production to determine EAI. Also, ESI was determined by taking 100 μl of the emulsion from the container bottom after 10 min. The aliquots were combined with 5 ml of SDS (0.1%), and the absorbance of the diluted solutions was read at 500 nm using a UV–Vis spectrophotometer. EAI and ESI were computed using the equations:

(7) $$\mathrm{EAI}\;\left(\frac{m^{2}}{g}\right)=\frac{\left(2\right)\left(2.303\right)\left(A_{0}\right)\left(\mathrm{DF}\right)}{\left(I\right)\left(\theta \right)\left(C\right)}$$

(8) $$\mathrm{ESI}\;\left(\min\right)=\frac{A_{0}}{∆A}∆t$$

where *A*_0_ is the absorbance of diluted emulsion at 500 nm immediately after homogenization, DF is the dilution factor (50), *I* is the path length of the cuvette (m), θ is the oil volume fraction (0.25), *C* is the protein concentration in the aqueous phase (g/m^3^), $∆A=A_{0}-A_{10}$, and ∆t=10min.

#### 2.12.3 Foaming properties

Two foaming properties including the foaming capacity (FC) and foaming stability (FS) of the CP, SP, and hydrolysates were assessed following the procedure reported by Rezvankhah, Emam‐Djomeh, et al. (2022) and Rezvankhah, Yarmand, et al. (2022). The 10 mg/ml sample solutions in a 50 ml measuring cylinder was whipped at 19,000 rpm for 2 min using a laboratory‐scale homogenizer (IKA, T25). The total volume (ml) of the initial foam was determined. Also, the foam volume was recorded after storage time of 30 min at room temperature. Foaming capacity and FS were calculated using the following equations:

(9) $$\mathrm{FC}\;\left(\%\right)=\frac{B-A}{A}\times 100$$

(10) $$\mathrm{FS}\;\left(\%\right)=\frac{C-A}{A}\times 100$$

where the volume before whipping (ml), the volume immediately after whipping (ml), and the volume after standing for 30 min (ml) are denoted by *A*, *B*, and *C*, respectively.

### 2.13 Statistical analysis

The experimental data were reported as means ± standard deviation. One‐way analysis of variance (ANOVA) was used to analyze the obtained data. The Duncan test was applied to evaluate the comparison of mean difference using the SPSS software (version 26, IBM software).

## 3 RESULTS AND DISCUSSION

### 3.1 Amino acid composition

Amino acid composition of the hydrolysates is presented in Table 1. The RP‐HPLC amino acid profile of CP showed higher hydrophobic amino acid contents, while the SP exhibited higher hydrophilic amino acid contents (Table 1). Similar findings have been reported in earlier investigations (Reyes Jara et al., 2018; Zhou et al., 2012). Enzymatic hydrolysis by Alcalase significantly changed the amino acid profiles of both proteins (Fadimu et al., 2021; Rezvankhah, Emam‐Djomeh, et al., 2022; Rezvankhah, Yarmand, et al., 2022). CPH had higher content of hydrophilic amino acids, while SPH showed higher content of hydrophobic amino acids than their respective native proteins (CP and SP) (Table 1).

**TABLE 1:** Total amino acid composition of CP, SP, CPH, SPH, SPH30:CPH70, SPH70:CPH30, and SPH50:CPH50 obtained by RP‐HPLC

Amino acid composition (g/100 g protein)	CP	CPH	SP	SPH	SPH30:CPH70	SPH70:CPH30	SPH50:CPH50
hydrophobicity							
Aspartic acid	5.49	6.07	10.93	12.36	7.92	10.52	10.08
Glutamic acid	21.99	24.53	16.36	27.07	25.28	26.16	25.27
Serine	4.35	4.82	4.31	4.82	4.95	5.0	4.75
Glycine	2.42	2.73	19.63	3.79	3.11	3.33	3.06
Histidine	2.09	2.24	2.01	2.45	2.10	2.54	2.43
Arginine	8.96	8.51	5.54	6.50	7.45	7.10	7.47
Threonine	2.93	3.06	3.59	3.52	3.21	3.47	3.34
Cysteine	2.71	2.19	0.64	0.74	1.56	0.99	1.53
Tyrosine	4.38	4.32	2.05	2.15	3.22	3.04	3.21
Lysine	1.32	1.80	7.00	8.19	3.72	6.09	5.03
	56.64	60.27	72.06	71.38	62.52	68.24	66.17
Hydrophobic							
Alanine	8.31	9.10	5.28	5.21	7.86	6.68	7.04
Proline	1.49	1.65	2.57	3.11	2.09	2.60	2.76
Valine	4.42	4.24	4.77	4.43	4.11	4.27	4.31
Methionine	3.88	3.51	1.20	1.08	2.58	1.83	2.59
Isoleucine	3.38	3.03	2.99	3.49	3.19	3.35	3.16
Leucine	16.09	13.83	8.73	8.23	11.92	9.77	10.28
Phenylalanine	5.78	4.38	2.40	2.87	5.72	3.23	3.69
Tryptophan	‐	‐	‐	‐	‐	‐	‐
	43.35	39.74	27.94	28.42	37.47	31.73	33.83

*Note*: CP and SP indicate unhydrolyzed protein of corn and soy, respectively. CPH, SPH, and different mixing ratio indicate hydrolysates of corn and soy, mixtures, respectively.

The variation in amino acid profiles could be related to the unfolding of the protein structures and exposure of the hydrophobic segments during enzymatic hydrolysis (Jin et al., 2015; Liu et al., 2012). This variation could also be due to the separation of some unhydrolyzed polypeptides during the hydrolysis and removal by centrifugation (Fadimu et al., 2021; Rezvankhah, Emam‐Djomeh, et al., 2022; Rezvankhah, Yarmand, et al., 2022). Furthermore, the amino acid composition varied among all mixed hydrolysates including SPH70:CPH30, SPH50:CPH50, and SPH30:CPH70. Indeed, the contents of hydrophilic and hydrophobic amino acids of the mixed hydrolysates were determined by the dominant hydrolysate portion. CPH was the major constituent in SPH30:CPH70, and the addition of SPH increased the quantity of hydrophilic amino acids when compared to CPH alone (Table 1). Previous studies have reported high hydrophobic and sulfur‐containing amino acid contents for CGM (CP) and CPH, and high hydrophilic amino acid and lysine contents for SP and SPH (Jin, Liu, et al., 2016; Jin, Ma, et al., 2016; Reyes Jara et al., 2018).

### 3.2 Surface hydrophobicity

**FIGURE 1:**
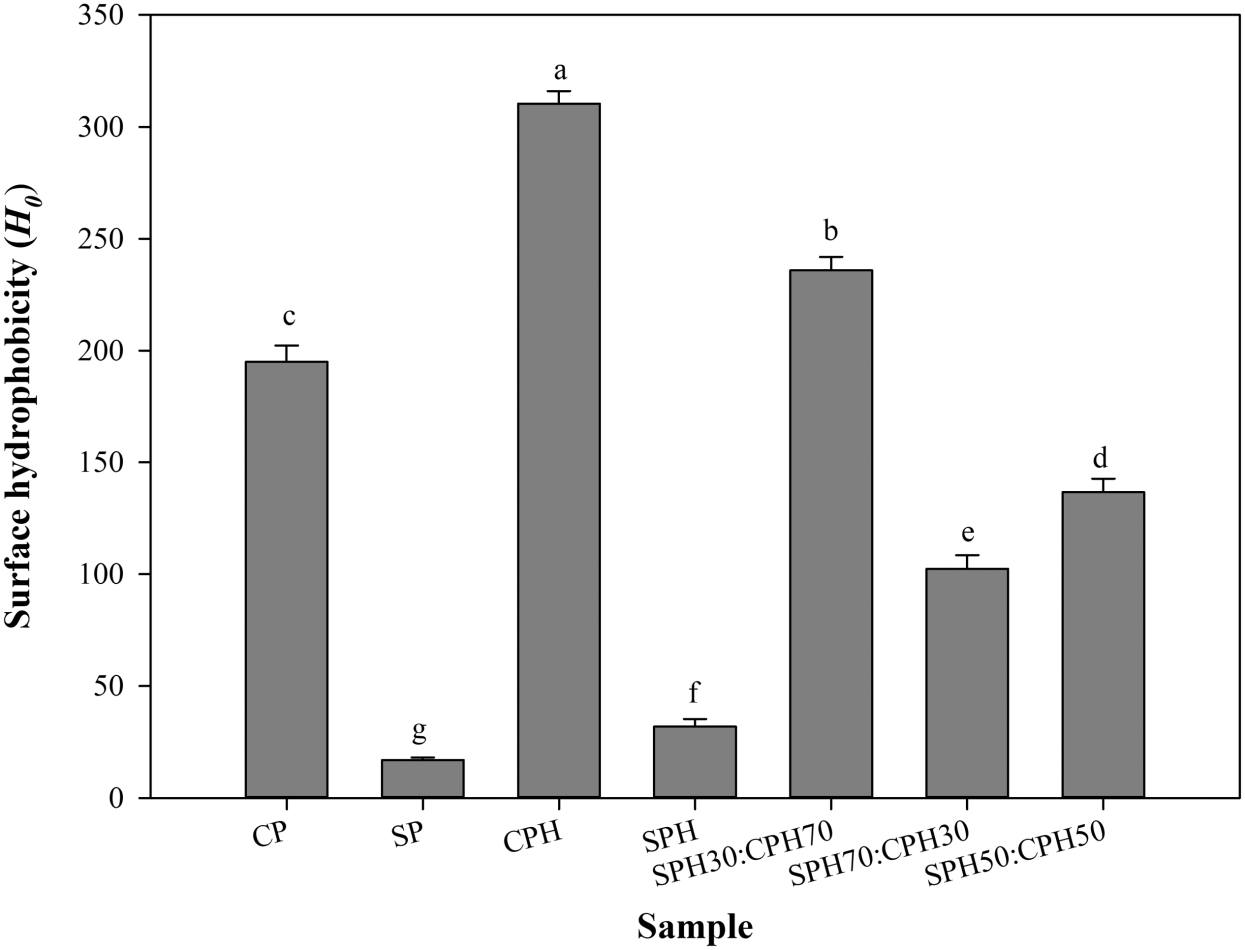
Surface hydrophobicity values of unhydrolyzed proteins, hydrolysates, and hydrolysate mixtures. The data marked with different letters are significantly different (*p* < .05). CP and SP indicate unhydrolyzed protein of corn and soy, respectively. CPH, SPH, and different mixing ratios indicate hydrolysates of corn and soy, mixtures, respectively.

Surface hydrophobicity (*H*_0_) has an important effect on the macromolecular structural stability, surface, and biological properties of proteins (Rezvankhah, Emam‐Djomeh, et al., 2022; Rezvankhah, Yarmand, et al., 2022; Wang et al., 2020). As shown in Figure 1, enzymatic hydrolysis of CP and SP significantly increased the *H*_0_ values, as previously reported by others (Zheng et al., 2015). The noncovalent, particularly the hydrophobic interactions, and disulfide bonds (SS) are abundantly present in CP (Liu et al., 2015; Wang et al., 2020). Although not considered the prevalent driving force for aggregation, hydrophobic interactions influence the aggregation tendency (Zheng et al., 2015). For CPH, the fluorescence intensity with ANS remarkably increased, indicating a higher *H*_0_ value than CP. The hydrophobic patches are buried inside the zein and glutelin structures (Liu et al., 2015). When the proteins are hydrolyzed, the hydrophobic segments are exposed to the surface. Albeit, it did not lead to aggregation. The insoluble aggregates may have been separated by centrifugation, while the soluble aggregates were maintained (Zheng et al., 2015). According to a previous study, CPH had an emulsion‐like appearance, which indicates that hydrolysis of CP not only increased its surface hydrophobicity but also decreased the MW and disulfide bonds of the protein, thereby transforming the insoluble aggregates into soluble aggregates (Zheng et al., 2015). Hydrolysis also increased the *H*_0_ of SP, but this increase in SPH was remarkably lower than CPH; this may be related to the amino acid composition of SP and CP or their corresponding hydrolysates (Rezvankhah, Emam‐Djomeh, et al., 2022; Rezvankhah, Yarmand, et al., 2022; Wang et al., 2016; Zheng et al., 2015) (Table 1). SPH had lower hydrophobic amino acid composition (28.42 g/100 g) than CPH (39.74 g/100 g), and this is likely due to the dominant hydrophobic amino acid portion in CP. As shown in Figure 1, the combination of SPH with CPH, depending on the dominant part, resulted in different *H*_0_ values. Therefore, the order of *H*_0_ values for combined hydrolysates of SP and CP was SPH30:CPH70 > SPH50:CPH50 > SPH70:CPH30. Therefore, the higher the content of CPH in the mixture, the higher the *H*_0_ value achieved (Figure 1).

### 3.3 Molecular weight profile

**FIGURE 2:**
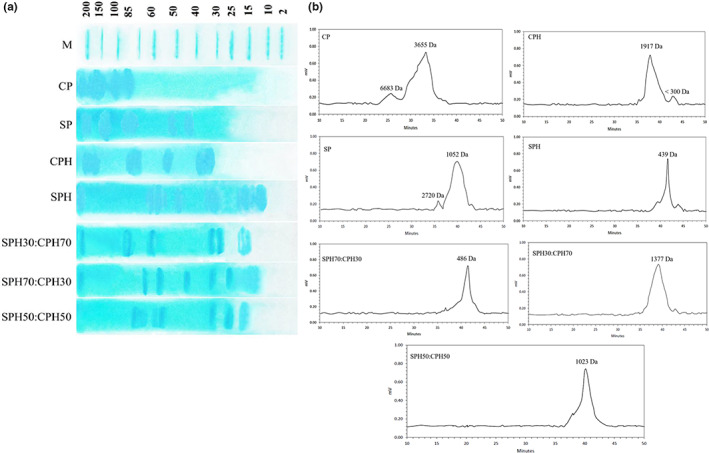
SDS‐PAGE patterns (a) and GPC MW distribution (b) of unhydrolyzed proteins, hydrolysates and hydrolysate mixtures. CP and SP indicate unhydrolyzed protein of corn and soy, respectively. CPH, SPH, and different mixing ratios indicate hydrolysates of corn and soy, mixtures, respectively.

Approximate MW of CP, SP, CPH, SPH, SPH30:CPH70, SPH70:CPH30, and SPH50:CPH50 was determined using SDS‐PAGE (Figure 2a). The intense bands detected for CP ranged from 80 to 200 kDa. A wide range of MW (250 Da to 250 kDa) have been reported for corn protein samples (He et al., 2021; Ortiz‐Martinez et al., 2017). For SP, the hydrolysates had MW of 40–50 and 70–200 kDa, the former indicating the presence of smaller polypeptides in SP than in CP. The electrophoretic pattern of SP showed β‐conglycinin subunits α′ (~72 kDa), α (~68 kDa), and β (~53 kDa), two subunits of glycinin, the acidic subunit (“A”) at 29–33 kDa and the basic subunit (“B”) at around 18–22 kDa (Meinlschmidt et al., 2016; Zhang et al., 2021). Hydrolysis of proteins significantly altered the bands that correspond to the smaller peptides produced and/or larger peptide cleavage by Alcalase (Jin, Liu, et al., 2016; Jin, Ma, et al., 2016; Liu et al., 2020; Xu et al., 2021). For CPH, bands with MW of 30–40, 50–55, 70–80, and 150–170 kDa were observed. CP did not show bands with MW <80 kDa, while the SDS‐PAGE pattern of CPH revealed polypeptides with MW ~30 kDa that was assigned to α‐zein, glutelin, and dimers (He et al., 2021; Liu et al., 2012). Indeed, zein, due to its low solubility, did not have a band at lower MW but proteolysis resulted in the emergence of peptides with higher solubility (Ortiz‐Martinez et al., 2017). For SPH, most of the SP bands at 70–200 kDa disappeared, and new bands were formed at 10–15, 15–20, 30, 40–50, and 50–60 kDa. The β‐conglycinin subunits α′ (~72 kDa) and α (~68 kDa), which are known as major soy allergens, completely disappeared after SP hydrolysis, as previously reported by Meinlschmidt et al. (2016). As shown in Figure 2a, the dominant portion (SPH or CPH) of the combination of the two hydrolysates determined the MW profiles of the mixture. For instance, the higher SPH ratio in SPH70:CPH30 resulted in the emergence of lanes mostly similar to the lanes detected for SPH, while the higher CPH caused the formation of bands specific to CPH (Figure 2a). The combination of SPH and CPH is hypothesized to present stronger biological activities and functional properties than their respective hydrolysates.

It has been reported that lower MW peptides are not visible on SDS‐PAGE gels, associating with heating effects on the protein conformation (Fadimu, Gill, et al., 2022; Rezvankhah, Emam‐Djomeh, et al., 2022; Rezvankhah, Yarmand, et al., 2022). Heating is performed for various aims (denaturation/unfolding of the protein structures and terminating of enzyme) during the hydrolysis stage or preparation of analytes for SDS‐PAGE analysis. Most of the SDS‐PAGE gels have been designed to determine molecules/peptides with MW above 10 kDa; thus, visualizing molecules with lower MW is possible using techniques such as gel permeation chromatography (Fadimu, Gill, et al., 2022; Rezvankhah, Emam‐Djomeh, et al., 2022; Rezvankhah, Yarmand, et al., 2022).

### 3.4 Molecular weight distribution

Changes in MW that could not be detected by SDS‐PAGE, especially MW below 10 kDa, were determined by GPC, given the high potential of GPC for accurate determination of MW distribution (Fadimu, Farahnaky, et al., 2022; Fadimu, Gill, et al., 2022). Molecular weight distribution of the proteins and their hydrolysates is shown in Figure 2b. Peptide size is one of the most important factors that influence the functional properties, bioavailability, and bioactivities of peptides (Rezvankhah et al., 2021a; Rezvankhah, Emam‐Djomeh, et al., 2022; Rezvankhah, Yarmand, et al., 2022). The chromatogram of CP showed short and sharp peaks assigned to MW 6683 and 3655 Da, respectively. The chromatogram of SP indicated short and sharp peaks with MW of 2720 and 1052 Da, respectively. These results as similar to previous findings with SDS‐PAGE patterns showing that SP had smaller polypeptides than CP (Zhang et al., 2021). As shown in Figure 2b, enzymatic hydrolysis resulted in the generation of small peptides with CPH showing a sharp peak at MW of 1917 Da and a short peak at MW of <300 Da. According to Figure 2b, SPH showed a peak for peptides with MW of 439 Da as previously reported (Wang et al., 2019). The combination of SPH and CPH, depending on the dominant portion, also altered the MW of the mixed hydrolysates. On this basis, the chromatograms of the SPH30:CPH70, SPH50:CPH50, and SPH70:CPH 30 showed sharp peaks at 1377, 1023, 486 Da, respectively (Figure 2b), indicating that the higher the SPH amount incorporated, the lower the MWs of hydrolysate mixture obtained.

### 3.5 Antioxidant activity

**FIGURE 3:**
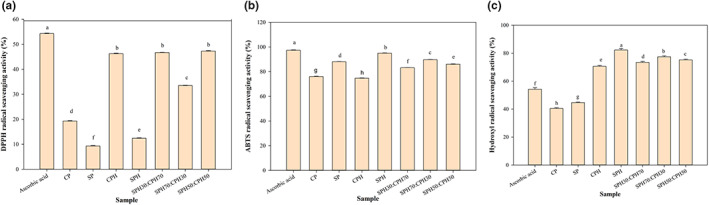
Antioxidant activity of unhydrolyzed proteins, hydrolysates, and hydrolysate mixtures. The small letters including (a), (b), and (c) illustrate DPPH, ABTS, and hydroxyl radical scavenging activities, respectively. Ascorbic acid (0.01 mg/ml) was used as a positive control. The data marked with different letters are significantly different (*p* < .05). CP and SP indicate unhydrolyzed protein of corn and soy, respectively. CPH, SPH, and different mixing ratios indicate hydrolysates of corn and soy, mixtures, respectively.

As shown in Figure 3a, among the proteins and hydrolysates, CPH (46.25%), SPH30:CPH70 (46.70%), and SPH50:CPH50 (47.30%) showed the highest DPPH RSA with no significant difference (*p* > .05), followed by SPH70:CPH30 (33.50%), CP (19.30%), SPH (12.40%), and SP (9.30%). Ascorbic acid, however, exhibited higher DPPH RSA at a much lower concentration than the hydrolysates. CP and its hydrolysates had high content of hydrophobic amino acids, which may be related to their higher reactivity with DPPH radicals (Jin et al., 2015; Karimi et al., 2020, 2021). Conversely, SP and SPH, which have high content of hydrophilic amino acids, showed lower antioxidant activity (Figure 3a). Enzymatic hydrolysis significantly increased the antioxidant activity of the proteins, and this is associated with the liberation of medium‐ and small‐sized peptides with exposed hydrophobic and reactive groups with antioxidant power (Zhou et al., 2017). CPH with higher hydrophobicity (Figure 1 and Table 1) had the strongest DPPH radical scavenging activity. Among all samples, SPH30:CPH70 and SPH50:CPH50 showed the strongest antioxidant activity.

A previous study on chickpea protein hydrolysates obtained with Alcalase showed that a content of over 50% hydrophobic amino acids resulted in high DPPH RSA (Quintero‐Soto et al., 2021).

ABTS and hydroxyl RSA gave different results. According to Figure 3b, all samples had ABTS RSA higher than 70%. The highest antioxidant power was obtained for SPH (95.01%), which was similar to the result of ascorbic acid (97.40%), followed by SPH70:CPH30 (89.70%), SP (88.03%), SPH50:CPH50 (86.04%), SPH30:CPH70 (83.22%), CP (76.07%), and CPH (74.75%). SP and SPH had higher reactivity with ABTS, while CP and CPH had lower reactivity, thus showing lower ABTS RSA. The production of peptides with MW <1 kDa remarkably influences the antioxidant power of hydrolysates (Tian et al., 2020). Also, SPH had higher hydrophilic amino acids that are known to have high interaction with the hydrophilic radical (ABTS) (Hu, Chen, et al., 2020; Hu, Dunmire, et al., 2020). ABTS radical has hydrophilic affinity, while DPPH radical has hydrophobic affinity; thus, SPH indicated higher ABTS RSA, while CPH exhibited higher DPPH RSA, similar to previous findings (Hu, Chen, et al., 2020; Rezvankhah, Yarmand, et al., 2022).

Based on the hydroxyl RSA results in Figure 3c, the lower the MW of the hydrolysates, the higher the antioxidant power obtained. SPH due to its higher hydrophilic amino acids and containing lower MW peptides (Figure 2a,b) exhibited higher hydroxyl RSA, while CP due to its higher hydrophobic amino acid profiles and larger peptides exhibited lower hydroxyl RSA (Rezvankhah, Emam‐Djomeh, et al., 2022; Rezvankhah, Yarmand, et al., 2022). MW of peptides can substantially affect their antioxidant activity. The small‐ and medium‐sized peptides have shown stronger antioxidant power due to their ability to interact with the radicals (Bu et al., 2020; Rezvankhah et al., 2021a; Singh et al., 2014; Tian et al., 2020; Zhao et al., 2021).

The order of hydroxyl RSA values was SPH (82.30%) > SPH70:CPH30 (77.40%) > SPH50:CPH50 (75.20%) > SPH30:CPH70 (73.40%) > CPH (70.60%) > ascorbic acid (54.20%) (as positive control) > SP (44.60%) > CP (40.50%), respectively. Taken together, the results suggest that SPH and CPH combined hydrolysate SPH70:CPH30 possessed the best antioxidant activities and, thus, have the potential to protect food or biological systems against oxidative damages. Zhang et al. (2021) reported potent hydroxyl RSA for SPH treated with ultrasound. Similar results were reported for mung bean protein hydrolysates (Liu et al., 2022). Also, CPH indicated potent hydroxyl RSA compared with nonhydrolyzed protein (Zheng et al., 2015).

### 3.6 In vitro antihypertensive property

**FIGURE 4:**
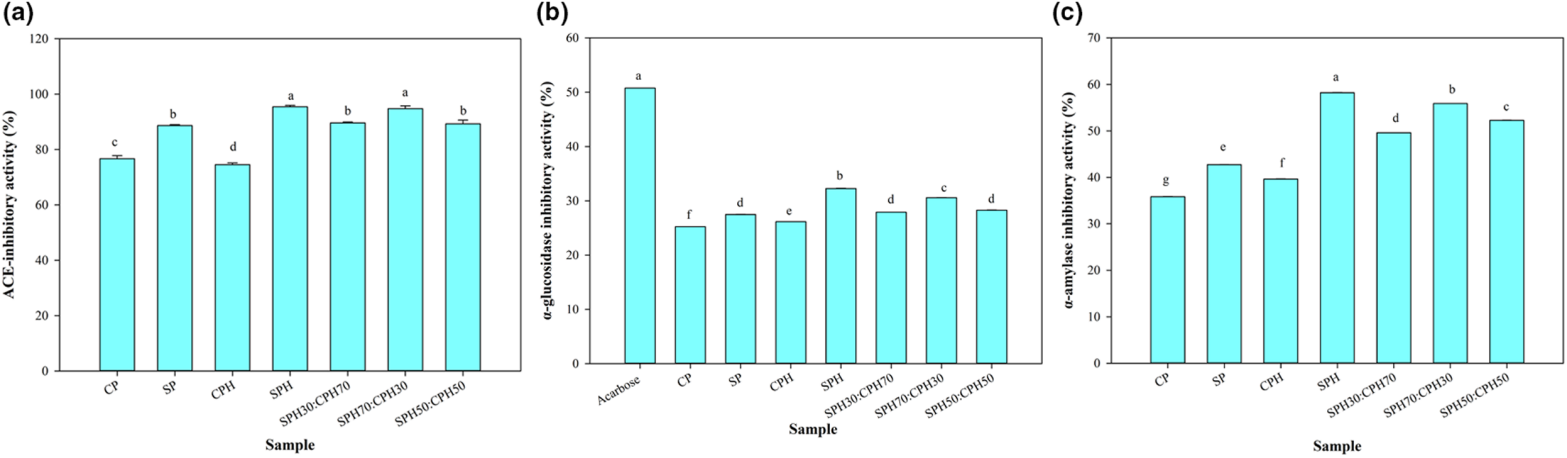
ACE (a), α‐glucosidase (b), and α‐amylase (c) inhibitory activities of unhydrolyzed proteins, hydrolysates and hydrolysate mixtures. The data marked with different letters are significantly different (*p* < .05). CP and SP indicate unhydrolyzed protein of corn and soy, respectively. CPH, SPH and different mixing ratios indicate hydrolysates of corn and soy, mixtures, respectively.

Results of the ACE inhibitory activity of the proteins and their hydrolysates are presented in Figure 4a. SPH exhibited the highest ACE inhibitory activity (95.45%) similar to SPH70:CPH30 (94.76%) with no significant difference, followed by SPH30:CPH70 (89.65%), SPH50:CPH50 (89.28%), and SP (88.64%), and the lowest values were obtained for CP (76.68%) and CPH (74.56%). Also, IC_50_ values of 0.5, 0.25, 0.38, 0.15, 0.23, 0.18, 0.21 mg/ml were obtained for CP, SP, CPH, SPH, SPH30:CPH70, SPH70:CPH30, and SPH50:CPH50, respectively. It was observed that hydrolysis of SP increased the ACE inhibitory activity as previously reported (Wang et al., 2019; Xu et al., 2021). Although proteins (SP and CP) and their hydrolysates (SPH and CPH) exhibited high ACE‐inhibitory activity (higher than 70%), the combined SPH and CPH also showed strong ACE‐inhibitory activity. The hydrophobic amino acids positioned at the C‐terminal residues have been shown to contribute to ACE‐inhibitory activity of peptides (Ambigaipalan et al., 2015). CP demonstrated weaker ACE inhibitory activity because the active peptide is locked in within the protein primary structure. Therefore, the activity obtained for the intact protein (CP and SP) could be due to unknown molecules co‐isolated with the proteins. Liu et al. (2020) identified 12 peptides from active fractions of Alcalase‐hydrolysate (CPH) obtained from CGM with good ABTS radical scavenging and ACE inhibitory activities (Liu et al., 2020). Similar findings were reported for SPH (Xu et al., 2021). Hence, the combination of CPH and SPH led to alterations in amino acid profiles of the new mixed hydrolysates, which influenced the bioactivities.

Angiotensin‐converting enzyme such as other enzymes has binding sites that could interact with peptide inhibitors (Quintero‐Soto et al., 2021). Higher interactions and affinity of peptides and ACE, indicated by lower binding energy, often result in stronger inhibitory activity (Quintero‐Soto et al., 2021). The hydrophobic amino acids located on the enzyme active site allow the interaction of uncharged amino acids.

### 3.7 In vitro antidiabetic properties

The in vitro α‐glucosidase and α‐amylase inhibitory activities of the samples are presented in Figure 4b,c. For α‐glucosidase inhibitory activity (Figure 4b), SPH showed the highest inhibition (32.26%), followed by SPH70:CPH30 (30.58%), SPH30:CPH70 (27.89%), SPH50:CPH50 (28.28%), and SP (27.48%) with no significant difference, and CPH (26.13%) and CP (25.20%) had the lowest values. The sample activities were lower than the effect of acarbose (50.76%). The IC_50_ values of 24.59, 13.51, 20.09, 5.65, 9.97, 7.15, 8.64, and 0.51 mg/ml were obtained for CP, SP, CPH, SPH, SPH30:CPH70, SPH70:CPH30, and SPH50:CPH50, and acarbose, respectively.

It was observed that the combination of SPH and CPH (SPH70:CPH30) led to mixed hydrolysates with higher α‐glucosidase inhibitory activity than CPH. Acarbose, a synthetic compound, at the same concentration exhibited much higher α‐glucosidase inhibitory activity than the crude hydrolysates. However, it has several side effects (Das et al., 2022), making the natural hydrolysates promising safer alternatives. The strongest α‐glucosidase activity of SPH could be associated with the higher content of smaller peptides produced at the same DH compared with CPH (Figure 2a,b). Also, the composition of amino acid residues influences the activity (Quintero‐Soto et al., 2021). The presence of the basic amino acids (lysine and arginine) at the end of the peptide chains and amino acids with hydroxyl groups (serine, threonine, and tyrosine) contributes to α‐glucosidase inhibition through the interaction with the active site of the enzyme (Karimi et al., 2020, 2021). The prevalent interactions are electrostatic and hydrogen bonds which lead to suppression of the enzyme activity (Rezvankhah, Emam‐Djomeh, et al., 2022; Rezvankhah, Yarmand, et al., 2022). Incorporation of SPH increased the α‐glucosidase inhibitory activity of CPH, likely due to augmentation of the amino acid composition.

As illustrated in Figure 4c, hydrolysis significantly increased the α‐amylase inhibitory activity of the samples. The highest α‐amylase inhibition was obtained for SPH (58.21%), followed by SPH70:CPH30 (55.88%), SPH50:CPH50 (52.26%), SPH30:CPH70 (49.57%), SP (42.74%), CPH (39.62%), and CP (35.85%). The IC_50_ values were 3.51, 1.22, 1.69, 0.23, 0.6, 0.33, 0.39 mg/ml for CP, SP, CPH, SPH, SPH30:CPH70, SPH70:CPH30, and SPH50:CPH50, respectively. According to Figure 4c, SPH and mixed hydrolysate with high contribution of SPH (SPH70:CPH30) showed higher α‐amylase inhibition than CPH and mixed hydrolysate with high contribution of CPH (SPH30:CPH70).

Bioactive peptides can interact with the active site of enzymes to reduce and/or inhibit substrate binding. Moreover, bioactive peptides can bind the allosteric site of the enzyme. For instance, peptides can interact with calcium and chloride ion binding sites of enzymes to produce unstable conformations, thereby restricting enzyme‐substrate binding (Ngoh & Gan, 2016). Indeed, calcium ions participate in structure formation, functions, and regulation of the stability of α‐amylase (Admassu et al., 2018). Moreover, amino acid residues including glycine, leucine, serine, aspartic and glutamic acids, proline, phenylalanine, tryptophan, and tyrosine have been shown to bind the active site of the enzyme, thus increasing the potential to achieve inhibition (Karimi et al., 2020). SPH and SPH70:CPH30 exhibited higher potential in inhibiting α‐amylase than the other hydrolysates.

### 3.8 Functional properties

Solubility, emulsifying, and foaming properties of the samples are provided in Table 2. The solubility of proteins and their hydrolysates was determined at pH 5, 7, and 9. Hydrolysis of CP and SP significantly improved the protein solubility. At pH 4, CP and SP were not soluble in water (0%) due to the insolubility of CP, zero net charges of the proteins (CP and SP), or lack of electrostatic repulsions (Rezvankhah et al., 2020; Zheng et al., 2015). Conversely, CPH, SPH, and their combinations exhibited higher solubility ranging from 92% to 96%. This result is related to the small‐sized peptides released during the hydrolysis process (Chen et al., 2011a, 2011b). At pH 7, CP and SP had 23.18% and 34% solubility in water, while CPH, SPH, and their combined hydrolysates exhibited higher solubility of 94%–100%. At pH 9, CP and SP had 24.50% and 96% solubility, which is likely related to the expected higher positive charge on the protein molecules. However, CP, due to its inherent low solubility in water (for zein and glutelin), still had low solubility at pH 9. Hydrolysis led to the solubility of 96% for CPH and 92%–100% for all hydrolysates.

**TABLE 2:** Functional properties of CP, SP, CPH, SPH, SPH30:CPH70, SPH70:CPH30, and SPH50:CPH50

Functional properties				
Sample	EAI (m^2^/g)	ESI (min)	FC (%)	FS (%) after 30 min
CP	12.43 ± 0.65^g^	07.60 ± 0.56^g^	00.00 ± 0.00^f^	00.00 ± 0.00^f^
SP	41.82 ± 0.52^c^	31.20 ± 0.41^c^	81.25 ± 6.25^b^	62.50 ± 3.50^a^
CPH	28.48 ± 0.16^f^	18.30 ± 0.42^f^	25.00 ± 2.10^e^	12.50 ± 1.30^d^
SPH	50.94 ± 0.91^b^	40.65 ± 0.91^b^	102.5 ± 5.50^a^	03.75 ± 1.25^e^
SPH30:CPH70	37.24 ± 0.48^d^	30.30 ± 0.14^d^	32.50 ± 1.25^d^	17.50 ± 1.20^c^
SPH70:CPH30	53.54 ± 0.62^a^	46.80 ± 0.70^a^	40.62 ± 3.12^c^	24.37 ± 0.62^b^
SPH50:CPH50	35.12 ± 0.87^e^	28.75 ± 0.63^e^	40.00 ± 1.25^c^	26.25 ± 1.25^b^
**Solubility (%)**				
**Sample**	**pH = 4**	**pH = 7**	**pH = 9**	
CP	ns^c^	23.18 ± 0.01^e^	24.50 ± 1.10^e^	
SP	ns^c^	34.00 ± 1.20^d^	96.00 ± 1.21^c^	
CPH	96.00 ± 2.30^a^	100.0 ± 0.04^a^	96.00 ± 1.11^c^	
SPH	92.30 ± 0.40^b^	94.00 ± 1.02^c^	92.00 ± 1.13^d^	
SPH30:CPH70	94.00 ± 0.30^a^	96.00 ± 1.20^b^	96.00 ± 1.30^c^	
SPH70:CPH30	94.00 ± 0.30^a^	96.00 ± 1.05^b^	98.00 ± 1.21^b^	
SPH50:CPH50	94.00 ± 0.30^a^	96.00 ± 1.10^b^	100.0 ± 1.42^a^	

*Note*: Different small letters in each column indicate significant differences (*p* < .05). CP and SP indicate unhydrolyzed protein of corn and soy, respectively. CPH, SPH, and different mixing ratio indicate hydrolysates of corn and soy, mixtures, respectively.

Abbreviation: ns, not soluble.

The emulsifying properties (EAI) of CP and SP were 12.43 and 41.82 m^2^/g, respectively, at pH 7 while their hydrolysates CPH and SPH exhibited significantly higher values of 28.48 and 50.94 m^2^/g, respectively. SP and SPH specifically had higher EAI values than CP and CPH. The mixed hydrolysates including SPH30:CPH70, SPH70:CPH30, and SPH50:CPH50 had EAI values of 37.24, 53.54, 35.12 m^2^/g, respectively. Notably, the highest EAI obtained for SPH70:CPH30 supported the hypothesis that a combination of 70% SPH with 30% CPH produces a synergistic effect. The EAI is associated with the protein/peptide ability to reduce the interfacial tension, thus generating smaller droplets leading to high emulsion stability (Rezvankhah et al., 2020, 2021b; Rezvankhah, Emam‐Djomeh, et al., 2022; Rezvankhah, Yarmand, et al., 2022). A similar trend was observed for ESI (min). Emulsion stability of CP and SP (7.60 and 31.20 min, respectively) significantly increased after enzymatic hydrolysis, with CPH and SPH showing ESI values of 18.30 and 40.65 min, respectively. This result could be related to the exposed hydrophobic regions, which can keep the oil at the oil–water interface. DH up to 15% improves both EAI and ESI of proteins due to an increase in water solubility and hydrophobic interactions (Wang et al., 2020). The mixed hydrolysates including SPH30:CPH70, SPH70:CPH30, and SPH50:CPH50 gave ESI values of 30.30, 46.80, and 28.75 min, respectively. Hence, the highest ESI value was obtained for SPH70:CPH30, indicating the strong surface‐active properties of SPH.

For foaming properties, CP showed FC (0%) while SP had a FC of 81.25%. Hydrolysis significantly increased the FC, which reached 25% and 102.5% for CPH and SPH, respectively. The improvement of FC could be related to an increase in solubility of the hydrolysates. SPH30:CPH70, SPH70:CPH30, and SPH50:CPH50 had FC values of 32.50%, 40.62%, and 40%, respectively. Limited hydrolysis not only enhances protein solubility but also increases hydrophobic interactions, thus increasing FC (Jin et al., 2015). FS showed a slightly different result (Table 2). Hydrolysis significantly increased FS for only CPH (12.5%). FS for SPH was significantly reduced, which might be related to the generation of small‐sized peptides with higher solubility, which influences the hydrophilic–hydrophobic balance. Interestingly, the mixed hydrolysates, including SPH30:CPH70, SPH70:SPH30, and SPH50:CPH50, showed higher FS than CPH and SPH. These results are likely due to the balanced peptide mixture in the combinations with optimum hydrophilic and hydrophobic interactions.

## 4 CONCLUSION

Hydrolysis of CP and SP changed the hydrophilic and hydrophobic amino acid contents of the resulting hydrolysates. CPH had higher hydrophobic amino acid contents, while SPH had higher hydrophilic amino acid contents. Consequently, the hydrophilic–hydrophobic amino acid ratio of SPH70:CPH30, SPH30:CPH70, and SPH50:CPH50 depended on the dominant hydrolysates in the mixture. The combination of CPH with SPH led to increase in DPPH RSA of SPH, and the combination of SPH with CPH led to the increase in ABTS and hydroxyl RSA of CPH. A similar trend was observed for surface hydrophobicity where combined hydrolysates with CPH showed higher surface hydrophobicity than SPH alone. SPH and SPH70:CPH30 had lower MW, and higher ACE, α‐glucosidase and α‐amylase inhibitory activities than CPH and SPH30:CPH70. SPH and CPH showed improved solubility, emulsifying activity, and foaming capacity. Furthermore, SPH70:CPH30 exhibited better functional properties than the other hydrolysates and mixtures. Considering all the biological and functional properties, SPH70:CPH30 can be further explored as a promising multifunctional ingredient with antioxidant, antihypertensive, antidiabetic, and functional properties for utilization in developing novel functional food products.

## FUNDING INFORMATION

This study was supported by the office of vice chancellor for research at Tarbiat Modares University (Grant Number: 9830422005).

## CONFLICT OF INTEREST

None of the authors declare a conflict of interest.

## ETHICAL APPROVAL

This study does not involve any human or animal testing.

## Data Availability

The data used in this paper are available in case of reviewer or editor request.
